# Youth’s narratives about family members smoking: parenting the parent- it’s not fair!

**DOI:** 10.1186/1471-2458-12-965

**Published:** 2012-11-12

**Authors:** Roberta L Woodgate, Christine M Kreklewetz

**Affiliations:** 1 Faculty of Nursing, University of Manitoba, 89 Curry Place, Winnipeg, MB, R3T 2N2, Canada

**Keywords:** Youth, Cigarette smoking, Second-hand smoke, Parents, Cancer prevention, Qualitative research

## Abstract

**Background:**

Successful cancer prevention policies and programming for youth must be based on a solid understanding of youth’s conceptualization of cancer and cancer prevention. Accordingly, a qualitative study examining youth’s perspectives of cancer and its prevention was undertaken. Not surprisingly, smoking (i.e., tobacco cigarette smoking) was one of the dominant lines of discourse in the youth’s narratives. This paper reports findings of how youth conceptualize smoking with attention to their perspectives on parental and family-related smoking issues and experiences.

**Methods:**

Seventy-five Canadian youth ranging in age from 11–19 years participated in the study. Six of the 75 youth had a history of smoking and 29 had parents with a history of smoking. Youth were involved in traditional ethnographic methods of interviewing and photovoice. Data analysis involved multiple levels of analysis congruent with ethnography.

**Results:**

Youth’s perspectives of parents and other family members’ cigarette smoking around them was salient as represented by the theme: *It’s not fair.* Youth struggled to make sense of why parents would smoke around their children and perceived their smoking as an unjust act. The theme was supported by four subthemes: *1) parenting the parent about the dangers of smoking; 2) the good/bad parent; 3) distancing family relationships; and 4) the prisoner*. Instead of being *talked to* about smoking it was more common for youth to share stories of *talking to* their parents about the dangers of smoking. Parents who did not smoke were seen by youth as the good parent, as opposed to the bad parent who smoked. Smoking was an agent that altered relationships with parents and other family members. Youth who lived in homes where they were exposed to cigarette smoke felt like a trapped prisoner.

**Conclusions:**

Further research is needed to investigate youth’s perceptions about parental cigarette smoking as well as possible linkages between youth exposed to second hand smoke in their home environment and emotional and lifestyle-related health difficulties. Results emphasize the relational impact of smoking when developing anti-tobacco and cancer prevention campaigns. Recognizing the potential toll that second-hand smoke can have on youth’s emotional well-being, health care professionals are encouraged to give youth positive messages in coping with their parents’ smoking behaviour.

## Background

Lung cancer is considered one of the most preventable types of cancer. While smoking (i.e., tobacco cigarette smoking) increases the risk of many forms of cancer, it is the predominant risk factor for lung cancer, accounting for about 80% of lung cancer cases in men and 50% in women worldwide [1]. Despite recent evidence that lung cancer is a high health risk concern to youth [2], adolescent smoking remains a public health problem. In 2010, 12% of Canadian youth aged 15 to 19 years smoked [3]. Although the number of youth smoking was the lowest level recorded for that age group, the decline in youth smoking rates has slowed [3] and youth smoking rates in some countries are rising [4]. In the United States, the surgeon general reported that more than 600,000 middle school students and 3 million high school students smoke cigarettes [5]. Also troubling is the fact that while smoking rates for youth are down, “of every three young smokers, only one will quit, and one of those remaining smokers will die from tobacco-related causes” ( [5], p. 9). Adult smokers frequently report having started smoking as youth. Among smokers aged 15–17, almost 80% said they had tried smoking by age 14 [6], with females (aged 15–17) having had their first cigarette at 12.9 years and males at age 13.3 years [7]. Global trends also reveal that smoking is increasing in developing countries due to adapting Western lifestyle habits. As well, lung cancer rates are increasing in some countries (e.g., China, Korea, and some African countries) and are expected to continue to rise for the next few decades [1].

Smoking has been shown to be a relational and learned behaviour, especially influenced by the family [8]. Regarding youth health behaviours, it has been suggested that the family, especially parents, is one of the dominant arenas in which youth are influenced. It has been established that youth are most likely to smoke if they have been exposed to, or come from a family in which their parents smoked [9]. Findings from a Canadian Community health survey revealed that in 2011 youth (aged 15–17) residing in households where someone smoked regularly were three times more likely to smoke (22.4% versus 7.0%) [10]. One qualitative study reported that aboriginal adolescent girls often smoke because smoking is normalized and reinforced by families: they see family members smoking in the home, they are not discouraged from smoking, and in some cases, parents facilitate adolescents’ access to cigarettes [11]. Youth smoking behaviour has also been linked to a range of other parental influences. For example, using a nationally United States representative sample, Powell and Chaloupka [12] studied specific parenting behaviours and the degree to which high school students felt their parents’ opinions about smoking influenced their decision to smoke. The authors identified that certain parenting practices (i.e., parental smoking, setting limits on youth’s free time, in-home smoking rules, quality and frequency of parent–child communication) as well as how much youth value their parents’ opinions about smoking, strongly influenced youth deciding to smoke.

The evidence indicates that while parents exert a strong influence on youth smoking, they can also exist as a protective factor against youth smoking [8,12-14], especially when non-smoking rules are in place [15] including eliminating smoking in the home [12]. Clark et al. [15] revealed that if parents themselves smoked, banning smoking in the home and speaking against smoking reduced the likelihood that youth will smoke. Similarly, other research on household smoking rules found fewer adolescent smoking behaviours in homes with strict anti-smoking rules [16]. While there is empirical evidence of how parents are greatly influential regarding their children’s smoking behaviours, we know little; however, about the dynamics involved in the parent-adolescent relationship regarding smoking in the home, or how youth perceive their parents’ approval or disproval of smoking behaviours. Few studies have addressed how children or youth feel about adult smoking.

In addition to findings that parental smoking may be related to the initiation of smoking in youth, there is increasing concern for the health risks of second-hand smoke. Along with anti-smoking legislation in public spaces, attention has been aimed at protecting children from second-hand smoke and recognizing the risks involved in exposure to second-hand smoke in non-public places. Second-hand smoking rates and non-smoking rules, for example, have been examined in family homes and cars [17-20]. In 2006, 22.1% of Canadian youth in grades 5 through 12 were exposed to smoking in their home on a daily or almost daily basis and 28.1% were exposed to smoking while riding in a car on a nearly weekly basis [19]. In the 2008 Canadian survey, the rate of exposure for 12–19 year olds (16.8%) was almost twice as high as the Canadian average [21]. New Zealand national surveys indicate that while exposure to second-hand smoke decreased since 2000, youth’s perceptions revealed that exposure still remained at 35% (in-home exposure) and 32% (in-vehicle exposure) [22]. The effects of parental smoking and maintaining a smoke-free environment has also received attention in areas such as prenatal and newborn care [23] and later, poor respiratory symptoms and outcomes [24]. Studies have also begun emphasizing home smoking bans and perceived dangers of the less visible but harmful exposure of third-hand smoke to children [23,25].

Although the current literature offers insights about the physical effects of second-hand smoke, how second-hand smoke impacts family relationships is unclear and what youth think about adult family members smoking remains in its infancy. This paper draws on data from a larger qualitative study that sought to extend our limited understanding of youth’s perspectives of cancer and cancer prevention. It aims to explore how youth conceptualize smoking within the context of their own life-situations with attention to their perspectives on parental and family-related smoking issues and experiences.

## Method

### Design

The qualitative research design of ethnography was utilized. Ethnography is the study of a specific cultural group of people that provides explanations of people’s thoughts, beliefs, and behaviours in a specific context with the aim of describing aspects of a phenomenon of the group [26-28]. For this study, youth were the group and the phenomenon of interest was youth’s perspectives of cancer and cancer prevention. Key assumptions that were integral to the successful undertaking of the study included viewing youth as self-reflective beings expert on their own experiences and as flexible agents existing within and being touched by multiple social and cultural contexts.

### Participants

Youth were recruited in a Western Canadian province from six schools in both a rural and urban setting. Schools mailed invitation letters about the study to families of potential participants who, if interested, could contact the researcher for further information. Purposive sampling techniques were used with the goal to achieve variation among participants based on demographic information (e.g., age, SES, gender, and urban or rural residency) and experiences in relation to cancer (i.e., some youth had family members who had experienced cancer). Recruitment ended once redundancy or theoretical saturation was achieved, that is, no new themes were apparent. In total, 75 youth ranging in age from 11 to 19 years (M = 14.5, SD = 2.1) participated in the study. The demographic and background characteristics of the participants are presented in Table 1.

**Table 1:** Demographic Profile of Youth Participants

**Characteristic**	**Category**	**N (N=75)**	**%**
**Age**	Mean = 14.5		
	SD = 2.1		
	Range = 11–19 years		
**Gender**	Male	20	27%
	Female	55	73%
**Ethnicity**	European	47	63%
	Canadian Aboriginal Canadian Aborigin Canadian Aboriginal	8	11%
	Other (Asian, African, Jewish, Arabic, Canadian)	14	18%
	Not Sure/No Response	6	8%
**Residential Situation**	Single parent household	13	17%
	Two parent household	53	71%
	Other (e.g. living with grandparents, step-parents, foster parents)	9	12%
**Geographic Location**	Urban	42	56%
	Rural	33	44%
**Income Status**	Low income (≤$35,000.00)	6	8%
	Middle income ($35,001.00-$70,000.00)	54	72%
	High income (>$70,001.00)	8	11%
	Not Sure/No Response	*7*	9%
**Family History of Cancer**	Parent with a cancer history	8	11%
	Sibling with a cancer history	3	4%
	Relative with a cancer history	11	15%
	No family history	53	70%

Of the 75 youth participating in the study, six (8%) had tried smoking but no longer smoked and four (5%) reported that they currently smoked. Twenty-two youth (29%) had parents who currently smoked, while seven (9%) had parents who had quit smoking. For the 10 youth (13%) who had a history of smoking, eight (11%) had parents who also had a history of smoking.

### Data collection

Data collection occurred between December 2007 and October 2010. The longer time period was due to school breaks and use of multiple data collection methods. The aim was to have each youth participate in two in-depth open-ended interviews. For the first interview an interview guide was used which included questions to elicit youth’s thoughts, beliefs, and feelings about cancer and cancer prevention (e.g., When you hear the word “cancer,” what does it make you think of? If developing cancer messages for youth, what would you tell them?). The interview guide had no direct questions about smoking. The open-ended nature of the interview guide provided an opportunity for youth to discuss areas they considered significant and/or areas previously not anticipated by the researchers [29].

After completing the first interview, all youth were asked to take part in the photovoice method. Photovoice is a participatory research method where individuals can address important issues through taking photographs and discussion [30-34]. Photographs, which are often used in ethnography, provided youth with a unique and creative means to reflect on cancer and cancer prevention. Youth were given a disposable camera to take pictures of people (with permission), objects, places or events that made them think of cancer and cancer prevention. Youth had four weeks to take photographs. During the second interview, participants were asked to talk about what the photos meant to them in terms of cancer and cancer prevention. In addition, youth were asked follow-up questions based on their initial interview and to comment on emerging themes. In total, 53 youth (71%) participated in the photovoice method and second interview. The remaining 22 youth (29%) were unable to complete the photovoice method and second interview due to scheduling difficulties.

Four focus group interviews with youth who were previously interviewed were conducted in the schools near the end of the study. The purpose was to identify ideas about cancer and cancer prevention that might emerge from a group context and provide quality controls on data collection [35-37]. Between three and four youth participated in each focus group. In total, fourteen youth attended the focus group discussions.

All individual and focus group interviews lasted from 60 to 90 minutes and were digitally recorded and transcribed verbatim. Field notes were recorded to describe the context (e.g., participant’s non-verbal behaviours, communication processes) and the interviewer’s perceptions of the interview. The interviews were conducted by trained research assistants under the supervision of the first author and took place at the participant’s school or home.

Finally, ethnographic field research was conducted. Research assistants were trained in fundamental skills and process in doing ethnographic field research including participant observation, field notes, and flexibility and openness [38-40]. On the days that research assistants were at the schools conducting interviews, they observed and recorded interactions and dialogue during informal daily activities (e.g., recess), special events (e.g., cancer prevention fund raising activities), and during the interviews themselves. Ongoing team meetings with research assistants allowed for debriefing and helped increase the sensitivity and richness of fieldnotes by critically highlighting features previously unconsidered by the observers alone. Raw field notes were written up and compared to interview data.

### Ethics

Before commencing the study, permission was obtained from the University of Manitoba Research Ethics Committee and from the recruitment sites. Parental consent and assent from all youth participants was also provided. Youth were informed that they could withdraw from the study at any stage. Strategies to secure the participants’ confidentiality were applied. Participants gave permission to use their photographs for the purposes of publishing and were reassured that any identifiable information in the photos would be removed (digitally altered). Youth received an honorarium gift card for their participation.

### Data analysis

Consistent with qualitative research design, data analysis occurred simultaneously with data collection. A data management system, NVivo version 9.0 [41] helped facilitate organization of substantial transcripts. Inductive coding began with RW reading all the field notes and interview transcripts. Analytical categories emerged from rigorous and systematic analysis of all forms of data (interview transcripts, ethnographic field notes, and photographs). Analysis of the data followed ethnographic principles of interpreting the meanings youth attributed to cancer and cancer prevention including their meanings attributed to parental and family-related smoking issues and experiences. Data analysis followed multi-level analytic coding procedures congruent with interpretive qualitative analysis and ethnography [28,29,39,40]. First-level analysis involved isolating concepts or patterns referred to as domains. Second-level analysis involved organizing domains. Through processes of comparing, contrasting, and integrating, items were organized, associated with other items, and linked into higher order patterns. The third level of analysis involved identifying attributes in each domain, and the last level involved discovering relationships among the domains to create themes. Various strategies were used to enhance the rigor of the research process including prolonged engagement with participants and data, careful line-by-line transcript analysis, and detailed memo writing throughout the research process [42]. The researchers independently identified theme areas then jointly refined and linked analytic themes and categories. Discussion of initial interpretations with the youth themselves occurred during the second interviews, which also helped reveal new data and support emerging themes.

## Results

**Figure 1:**
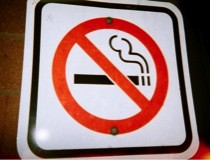
**Anti-Smoking Sign.** Represented youth’s desire that adults should stop smoking.

Smoking was one of the dominant lines of discourse across the sample of youth’s narratives of cancer and cancer prevention. Age, gender, smoking status (i.e., smoker or non-smoker), and place of residency did not influence the story line. Youth were considerably more knowledgeable about the association between smoking and cancer and anti-tobacco messages in comparison to any other cancer-related topic. Several youth photographed their own hand-drawn facsimiles or public signage depicting familiar anti-smoking slogans (Figure 1).

"When I walk around there’s like no smoking area signs. I think “Oh this is safe,” like it is good to know that there won’t be like smoke around for me to breathe in. [14-year-old male]"

Youth in this study were well informed of how smoking could impact one’s health (e.g., increased the potential for cancer and other chronic illnesses). Of special importance were youth’s perspectives and experiences of parents and other family members smoking around them as represented by the primary theme, *It’s not fair*, and four subthemes*: parenting the parent about the dangers of smoking; the good/bad parent; distancing family relationships; and the prisoner.* Each of these themes are further discussed.

### It’s not fair

Overall, youth viewed their parents and other family members smoking around children as something unjust. The phrase “It’s not fair” was frequently expressed by youth in this study as illustrated in the following comment,

"Because the kids around parents who smoke have to breathe in, they have to breathe in all of it …and like, if parents want to smoke then they should like go outside because***it’s not fair to the kids…***Probably because they always have to be around it if their mother always smokes every time they’re taking a bath or every time they’re like colouring a picture like every time they do anything, they always have to breathe in the bad- like bad air that’s filled with smoke and stuff like that. And***it’s not fair***to them. [12-year-old female]"

Youth could not make sense of why parents would smoke around their children. They also were unsure with how to deal with what they saw was an act of injustice to children. They struggled about how the smoking made them feel, recognizing that their roles as children limited their ability to influence their parents’ behaviour. Their attempts to reconcile their feelings and deal with the unfairness through specific behaviours are further apparent in the following four subthemes.

### Parenting the parent about the dangers of smoking

Although youth did share stories of parents *talking to them* about the importance of not smoking, this was not the major family narrative. Instead of being *talked to* about smoking, it was more common for youth to share stories of they themselves *talking to, educating, and even preaching* to their parents about the dangers of smoking. In short, youth took it upon themselves to parent their parents.

"But I’m getting my mom and my step-dad to quit…by talking to them, telling them how it makes all of us kids feel…Yeah, reading like everything the packages say or what the internet says or like what I learn from it and then they’re all just thinking and then they’re saying “well I won’t do that much then. I’ll try to quit.” Now they’re trying to quit but it’s not working for my mom but it is working for my step-dad. [14-year-old female]"

In addition to talking to their parents about how they felt about them smoking, some youth also would take action to reduce their parents’ ability to smoke.

"Like I have just tried, because I just tell my parents straight up to stop and…I always try to, like, hide their stuff on them but it doesn’t work. They get mad. [13-year-old male]"

**Figure 2:**
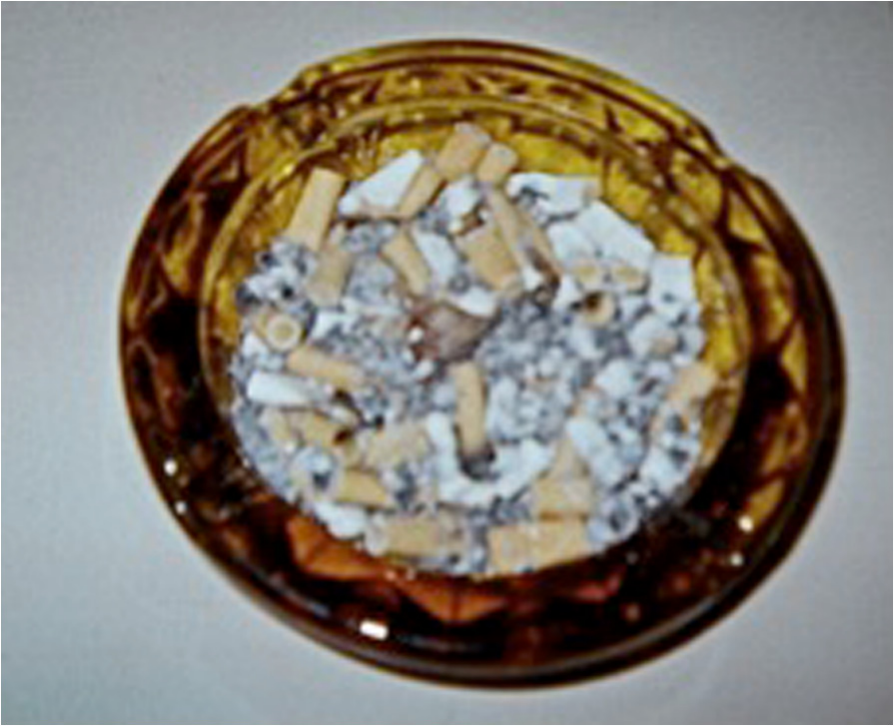
**Ashtray.** Represented youth’s concern for parents.

Talking to their parents about the dangers of smoking arose out of youth’s worries for their parents’ health. The concern that youth had for parents who smoked was in fact one of the reasons youth decided to participate in the study. Youth were looking for answers that could possibly help them to get their parents to stop smoking. Concerns about their parents smoking was also strongly depicted in the photographs. One 13-year-old female took a picture of an ashtray full of cigarettes and said,

*"I see them (ashtrays with cigarette butts) all the time. It would be different if it was like you know once in a while kind of thing I probably wouldn’t mind that much, but my parents smoke in the house and in the car and everywhere so it’s kind of I don’t know I wish they would stop* (Figure 2).*"*

Youth who feared that their family members might die, or who had family members who died because of smoking-related illnesses (e.g., lung cancer), especially shared their concerns and would try to make their parents feel guilty.

"I always tell them things to make them guilty I’m always like, “Do you want to meet my children, do you want to bring me down the aisle?” It’s working actually. I think my dad said my mom was talking about it so…Ever since my mom started again I really felt it hit me cause, like I want my mom to meet my children and she has to see me get married and have kids and I want my mom to be there. [16-year-old female]"

Youth also provided many stories of their parents’ attempts to quit smoking. Their stories demonstrated how smoking was embedded within their family’s history and identity, as well as how parents’ smoking played a role in their child’s life.

"Well I was really happy when my father quit because he had been smoking since like, I don’t know, before he was even a teenager. He was really young and he said he’d quit sort of when I was born like he’d smoke outside and he’d reduce it, but then when I got old enough he’d continued smoking, and my mom was the same way. She was also a smoker, but she quit like maybe I was five… [15-year-old female]"

"My grandpa passed away a couple of years ago and he died of emphysema and a little bit after he got sick I should say he quit smoking and whenever my aunties and mom smoked, but they don’t anymore, then he would always tell them “Well you should quit because look what’s happening to me.” And that really pushed my mom to quit. [17-year-old female]"

While youth were persistent in trying to convince their parents to stop smoking, most youth felt that their parents would not quit despite their efforts. A sense of helplessness was apparent.

"Well, my parents like they are smoking and if I tell them not to they’re not going to listen because they’re like “We are your parents, you’re not our parents!” [18-year-old male]"

"Like my mom and my dad both smoke and I’d like to tell them to stop and to show them…they don’t really care…I don’t know, like influencing somebody not to smoke is a lot different than I guess them already smoking and quitting. Because like, I mean obviously everything I’ve seen I’m never going to smoke, but I mean it doesn’t really influence my parents. [13-year-old male]"

### The good/bad parent

A second sub-theme involved a moral tone in youth’s conversations with respect to how they viewed their parents and other family members who did or did not smoke. On one hand, youth perceived parents who did not smoke as *doing the right thing* and as part of their parents’ overall plan of keeping themselves and their children healthy.

"Like my parents don’t smoke, they don’t like do drugs or anything like that and they do like everything possible to stay to like be healthy and stuff and to keep me healthy and stuff like that. [12-year-old female]"

In contrast, youth were especially disapproving of parents who did smoke.

"Like if a mother wanted to have a baby so badly in the first place then she should have known that she’s not supposed to drink or she’s not supposed to smoke or she’s not supposed to do any type of drugs or anything…and they don’t know how bad it actually is for the kids who have to breathe it in. [14-year-old female]"

Parents’ second-hand smoking was seen by youth as parents “doing” to their children. “Doing” was viewed in a negative sense where children were put in a dangerous situation in which they had little choice or control as depicted by the following quote and photo.

**Figure 3:**
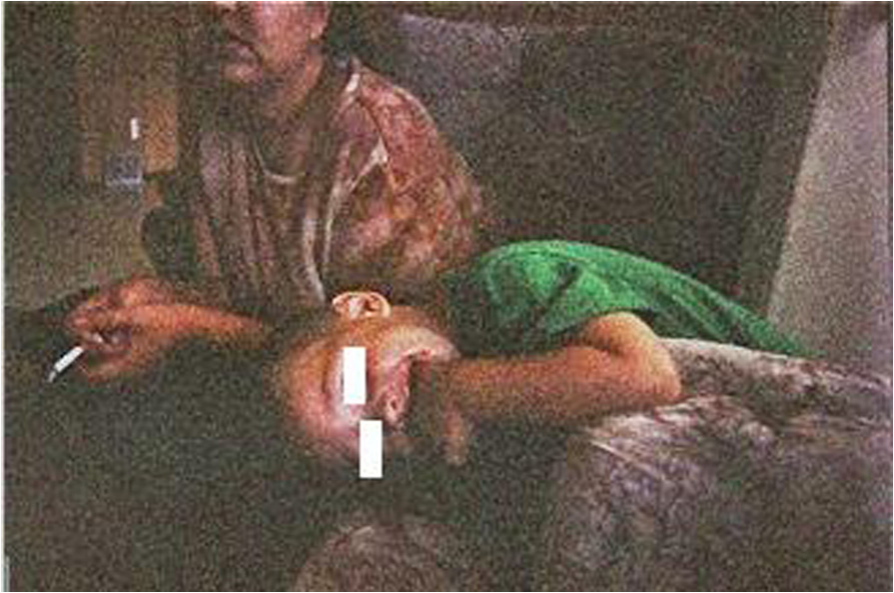
**Adult smoking beside a young child.** Represented parents “doing” to children; children have no choice.

*"I guess for people with families already, like what it’s doing to their family or that second-hand smoking can be almost as bad as actually smoking like what are you doing to the people around you if you’re choosing to smoke.* [17-year-old female] (Figure 3).*"*

Essentially, youth felt that second-hand smoke was more dangerous than first-hand smoke as one 15-year-old male noted, *“This stuff (second-hand smoke) does not get filtered through the back of the butt, it just comes out clear not filtered.”*

Youth expressed concern for how second-hand smoking impacted them and their siblings.

"Both my parents smoke so I don’t like it too much because the smell is kind of it bugs me and you know I don’t know because so many people talk about smoking is related to cancer and that kind of thing so I’m kind of scared. I’m scared for myself and for my parents. But I’m more scared for like my brother than I am for me because I can leave more often than my brother’s allowed to… so. Either like my brother developing the habit or something or like him getting cancer because he’s around it too much. [13-year-old female]"

Many youth, whose parents did not smoke currently or had never smoked, were concerned about the effects of second-hand smoke on their friends (whose parents smoked). These youth spoke about their friends’ situations vicariously. Their comments in the interviews arose from their extended empathy towards their peers and their peers’ siblings.

"Um, I’m pet sitting for a friend while she’s in Florida and her parents are usually always smoking or getting ready to light another cigarette and so I went there it just smells so bad in their house and I feel sorry for her cause she’s got a sister in kindergarten and it’s her in grade nine and her parents are smoking and their dogs in there and cat and it just sticks to the furniture and it just smells smoke. [14-year-old female]"

Youth felt that parents who smoked were poor role models and that their behaviour could influence their child’s desire to smoke.

"Cause when children see, children do, right? Yeah, so lots of kids when your parents smoke when you’re like in grade two or something and kids get the idea that it’s cool or like whatever and then they want to be like their parents cause they think their parents are awesome. So then they start doing everything their parents do and then they start smoking… [14-year-old female]"

"Or sometimes you can get addicted to smoking if both your parents smoke a lot and then like my cousin who smokes uses that excuse cause I’m like”Why do you smoke, that’s disgusting!” And he says “Well both my parents smoke so I started.” I don’t think his parents are a very good influence since they both smoke. And I’m not sure if they ever told him not to smoke, but maybe they just accepted his smoking not saying it is bad or anything. Like if my kid started smoking I would get mad. [13-year-old male]"

Parents who smoked were also considered by youth to be less reliable and credible when talking to their children about the dangers of smoking.

"When my parents found out I tried it (smoking) once, they knew that they couldn’t do anything cause since they were smoking too! [13-year-old female]"

In general, parents and other adult family members who smoked were viewed by youth to be weak in character.

"So like one year he (family relative) came out from Ontario with his wife and my grandma and it was pouring rain and he decided to go outside for a smoke, so he really ran across our yard and hid in the shed and smoked. I’m like, it’s pathetic. [14-year-old female]"

It was evident from the youth’s narratives that parents’ smoking behaviours were unacceptable and should never be tolerated.

"And I mean people really need to kind of jump on it and say don’t do this around your kids because it will affect them. Don’t do this around any young child because young children are really open to being affected by something like that and so I think definitely being careful about where you’re smoking or something like that is definitely a really big factor. [16-year-old female]"

### Distancing family relationships

A third sub-theme that emerged was one where youth were separating themselves, both physically and emotionally, from their family members. In addition, youth associated smoking with causing emotional stress or strain on the family.

"***Youth:***And they always get problems because of it so I kind of don’t want to have to deal with all those problems. And all that stress and everything so I’m just going to like leave it alone.***Interviewer:***Okay. So like what problems?***Youth:***Like family issues.***Interviewer:***So like you said that they have family issues and stuff, why is it important to you not to have that in your life like those things?***P:***Cause I already have enough family problems. I guess I don’t want anymore. [14-year-old female]"

The discussion with youth revealed that smoking had, in varying degrees, disrupted family relationships. Just the presence of family members smoking around them resulted in youth altering their behaviour and wanting to physically distance themselves from smoker family members.

"I live with my grandparents. They make me supper and then I have to usually eat in my room because they smoke, and I don’t like the smell of smoke when I’m eating. I hate the smell and it’s just I grew up my whole life with it and I just think I just see my grandma a lot of my family members have like my great-grandma had passed away with lung cancer and stuff. I just think it’s bad. [17-year-old female]"

At times, being around parents who smoked resulted in feelings of worry and frustration for youth.

"Well my step-dad smokes and he’s always saying, “No, it won’t happen to me, it won’t happen to me!” And he actually has a really high chance of catching it cause he started smoking when he was really young and he continued smoking and, uh, he still thinks it won’t happen and doesn’t believe any of the commercials or the ads on that stuff. He just keeps going so… [17-year-old male]"

"I was riding with my dad and he was smoking. I was like, “Do you have to do that when we’re in the car?” Like I get so bugged by it when people do it. It’s like. “Look at the cigarette box!” I get so mad. I was like…like when we were getting out of the car and I said, “Oh can you just not do that?” I walked ahead of him and he said, “I’m sorry.” It’s like, “Okay!” [15-year-old female]"

Youth were also sensitive how their negative reactions and behaviours towards family members who smoked could result in hurt feelings.

"Whenever they smoke around me I just like take a shirt or something and just like cover my mouth and nose and my brothers and sisters are doing that too. Yeah, so just to try and keep it away. My parents don’t mind, but I’m pretty sure it hurts their feelings or something. [13-year-old female]"

Many youth described family tension and conflict because a family member was smoking.

"It can really bring your family down, if smoking hurts someone in your family. Um, it could really cause a lot of tension there… [16-year-old male]"

"And like my cousin, her parents smoke. They quit. She helped them quit but then they started again and then she started crying and crying and crying and crying, and then she’s scared that her parents are going to die from lung disease or a lung cancer and she always cries when they do that and then one time they said “It’s our choice if we do this. It’s not your fault if we die or not and they said that they only smoke once a day, not too much.” Now she’s still gets mad but she doesn’t have temper tantrums anymore. [12-year-old female]"

Feelings of anger were also associated with family members who smoked. One youth who had an extended family member who died from lung cancer was upset with a son-in-law who continued to smoke in spite of his father-in-law’s death.

"I saw him smoking and I was like “Why? Like you saw what your father-in-law went through. Why are you still doing that?!” And it, it really angers me. Like I don’t know, just even talking about it gets me so mad like you’re seeing all these things like you know it happens. Why are you going to ruin that? [15-year-old female]"

**Figure 4:**
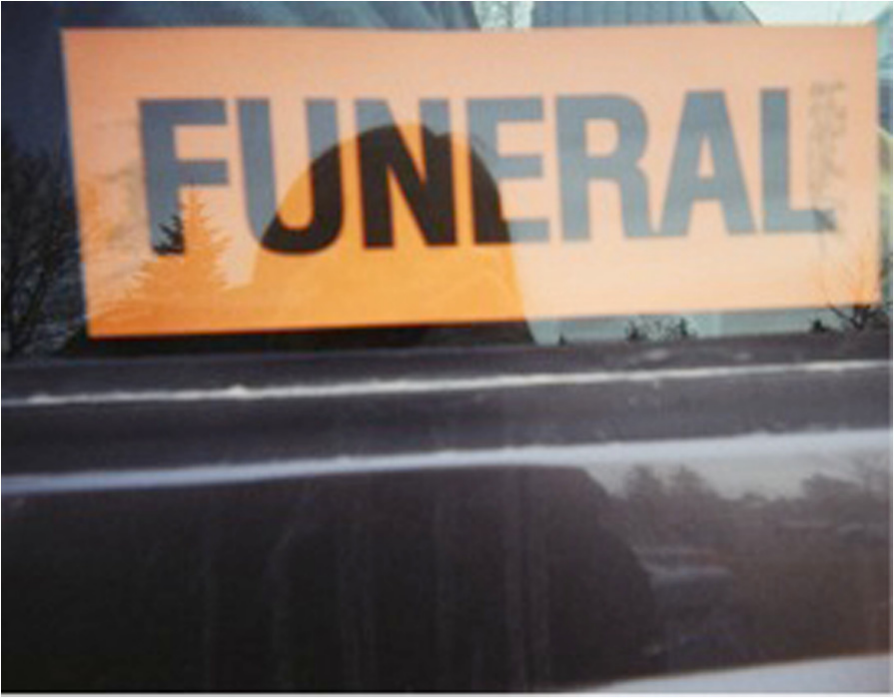
**Funeral Sign.** Represented smoking as a sign of cancer and death.

Notably, of all the feelings expressed by youth in the study, it was a deep sense of sadness that was most apparent in their narratives with respect to families who had a history of smoking. The sadness was in relation to a past or future loss of family members. For some youth, the sadness was physically evident through youth crying and holding back tears while being interviewed. Some held the view that smoking was the defining feature of their families that ultimately would lead to its destruction (Figure 4).

"Lots of my family smokes and I’m worried about them getting cancer and then not surviving it. [16-year-old female]"

"Okay, I took two pictures of smokes cause the first reinforces that smoking could lead to lung cancer. And the second is cause it relates to me and my life because my mom smokes. My grandpa smokes, my grandma smokes, my aunty smokes because a lot of people smoke in my family. Well I feel sad that she probably could die soon like she maybe diagnosed with cancer like any time because she smokes a lot. [13-year-old male]"

### The prisoner

The final sub-theme that emerged in the study was a sense of resigned acceptance, powerlessness, and being held as a prisoner. Ultimately, the unjust nature of parents smoking in the family home was truly felt by those youth who described having little choice but to feel trapped inside the smoke.

"Um, yeah. Well I had to stop volleyball and taekwondo for a little while because my knees were really bad and I have been experiencing like a hard time breathing. But I think that’s particularly because after my dad died my mom let these people move in and the guy smoked a lot and I wasn’t used to that amount of smoke in my personal area. Like downstairs was all mine before, but then I was close to my bedroom and his smoke would come in my room. So I was I was stuck with that all the time. [16-year-old female]"

"Whenever he smokes I’m like in a car with him or in the house with him. He’s always supposed to go outside of the house to smoke, but when I’m in the car with him I roll down the windows so I don’t have to breath in the smoke and I just go on with him and like, “Okay, you can do whatever you want, then I’m just going to do what I want to do.” [17-year-old male]"

Within their home (and while travelling in vehicles with their parents), youth had few ways of escaping the second-hand smoke and little, if any, influence over their parents’ smoking behaviours and rights to live in a pollution-free environment. Some even described how they had to cover their nose and mouth when walking through their house. These youth were like prisoners within their homes. They experienced their own, and witnessed their siblings’ exposure to second-hand smoke, but felt they were unable to help and protect themselves, let alone their siblings. Youth expressed feeling caught in an unpleasant situation which was difficult to escape. They perceived it as unfair and just had to put up with it.

## Discussion

Across the sample, smoking was one of the dominant lines of discourse in the youth’s narratives of cancer and cancer prevention. This is not surprising and is consistent with a recent study examining perceived smoking related adverse effects, where youth consistently rated lung cancer as being most concerning [2]. It appears that youth are connecting smoking with cancer risk. Youth’s discourse (as reinforced through their photographs) reflected the broad public health messages conveyed by anti-tobacco and cancer prevention campaigns suggesting that youth are not passive and ill-informed with respect to tobacco use and health messages. Instead of being preached to by parents about the dangers of smoking, it was youth themselves who were speaking out against smoking. To date, very few studies have reported such behaviour.

Ours is one of few studies that detailed youth perceptions of parental smoking and second-hand smoke and their association with health concerns and family relationships. Although there is research indicating that youth believe parents have an obligation to do all that they can to support their children to not start smoking [13], this study revealed that youth are taking responsibility by parenting the parent about the dangers of smoking. Youth in this study demonstrated a high awareness of the dangers of smoking. They expressed fear and concern about the health-related effects of smoking, especially regarding second-hand smoke in their families. Regardless if there was a history of smoking in their families, all youth were worried about the dangers of second-hand smoking for themselves and other family members. Moreover, their awareness and concern extended beyond their own family unit.

Our study supports previous research reinforcing that youth continue to be frequently exposed to second-hand smoke in their homes and in cars even though most youth do not approve of it [19]. Overall, youth viewed the smoking behaviours of significant adults in their lives as an unjust act that all adults should be aware of. Although the concern for protecting children from second-hand smoke should be about recognizing children's rights over adult smoker's rights, research has shown that the responses of adults to smoking bans in homes and cars have not always been met with compliance and acceptance [17,18]. In a study that investigated New Zealand policymakers’ views on the regulation of smoking in private and public places, findings revealed that policymakers were more apt to defer to a smoker’s right to smoke, rather than the protection of children from second-hand smoke [43]. In that same study, some participants suggested that the successful regulation of smoking around children in private places will require reconstructing the culture around smoking in which any smoking around children would be considered unacceptable.

Our current study adds to the literature as it identifies the various strategies youth use to deal with second-hand smoke in their home environments. In addressing second-hand smoking by their parents and other family members, youth opposed their parents’ smoking behaviours verbally or through their body language. Youth tried to cope with the situation in a variety of ways such as distancing themselves from family members, eating meals in their rooms, smoking with family members, and physically covering their faces to avoid exposure of second-hand smoke. However, despite their attempts, youth were forced to accept the fact that smoking was a defining feature of their families. Results suggest a great toll on youth’s emotional state and their ability to cope with their parents’ health compromising behaviours.

Although research discusses how parents’ behaviours, related to smoking, influence the behaviour of youth, there is minimal discussion on how youth view the impact of smoking on the family unit. Through using qualitative methods, this study showed how smoking was an agent that altered youth’s relationships with family members, usually in a negative way. This is in contrast to a qualitative study by Passey and colleagues that explored the social context of smoking among aboriginal women and girls [8]. The researchers found that for young girls, smoking was an important way of maintaining relationships within the extended family and community. Sharing a cigarette was seen as a social activity within the family that built a sense of belonging. However, in our study, smoking behaviours of parents and other family members tended to produce feelings of concern, hurt, resentment, and a detachment from the family. The positive or negative impact that cigarette smoking can have on relationships, suggests there is a strong relational component to the act of smoking.

While the focus on the dangers of second-hand smoke has mainly been on the physical health of children, our study reinforces how the harmful effects of parental smoking also extend to youth’s emotional well-being. Overall, youth reported experiencing stress, worry, helplessness, anxiety, and fear for their health and the health of friends whose parents smoked. These youth experienced a heavy emotional burden to *parent the parent* and carry concerns about siblings. Youth living in families where adult members smoke are least likely to have control over whether they are exposed to second-hand smoke; this places them not only at risk for physical health problems but for emotional distress as well. This study calls attention to the need for future research exploring how the emotional well-being of youth living in homes with adults who smoke, may go dismissed or unrecognized.

Although research has examined some of the health-related effects of having substance-abusing parents, it has for the most part overlooked the detrimental effects on children and family functioning where parents use more socially “acceptable” addictive substances such as tobacco and nicotine. The literature is robust in its findings about the negative effects of living with a parent who has an addiction to alcohol. For example, adolescents of alcoholic parents reported mental health difficulties (including emotional symptoms) and other behaviours (e.g., academic performance and conduct problems) compared to a control group [44]. Children growing up in substance-abusing families have been shown to have a disrupted family life with increased family conflict, and may be at greater risk for developing alcohol, drug-related, and behavioural problems [45].

Tobacco addiction is a major public health concern. The findings emerging from this study reinforce the need for public health action in three areas. First, more public health-related research is warranted that examines youth’s perceptions about their life circumstances growing up in families where their parents smoke. Further research is needed to investigate possible linkages between youth exposed to second hand smoke in their home environment and emotional and lifestyle-related health difficulties.

Research is also needed to investigate how youth’s perceptions about being exposed to parental smoking behaviours and second-hand smoke impact on family relations and youth development. A report by Children’s Mental Health Policy Research Program at a Canadian University reinforces that research evidence on children's mental health needs may be best informed and strengthened by the participation and experiences of children and their families [46]. Creating a scientific base on youth’s perspectives of their health and well-being in the context of youth living with parents who smoke, is a critical step to improving and supporting youth's physical, mental, and emotional health.

Second, the findings also raise the issue of attending to the emotional well-being as well as the physical needs of children who reside in smoking households. We need to assist youth who live with second-hand smoke in their homes, and who worry about the health effects on their parents and other family members. Public health programs and policies that help to empower youth who live in families in which parents and other family members smoke are needed. Song et al. recommend that encouraging youth to express their objections to second-hand smoke, as well as encouraging smoke-free homes, may be powerful tobacco control strategies against youth smoking [47]. In addition to controlling smoking within households, the findings from this study may be used to move forward tobacco control programs and policies designed to prevent parents and other adults from smoking around youth in locations outside the home where parents and youth interact. A comprehensive tobacco control program should support the need for more smoke-free public places including patios, playgrounds, sports fields, beaches, provincial parks, public events, and building perimeters [48].

Upon recognizing the potential toll that parental smoking can have on youth’s emotional well-being, community-based programs to help youth experiencing stress due to their concerns about the dangers of second-hand smoke are needed. Few supports are provided for youth of tobacco-addicted parents, especially for non-smoker youth who are experiencing distress because of their parents’ self-harming behaviours. Health care professionals also can be encouraged to give youth positive messages in dealing and coping with their parents’ smoking behaviour. Addressing youth’s concerns and distress related to second-hand smoke is essential for youth to thrive both physically and mentally.

A final area for public health action is that anti-tobacco messaging to adult smokers needs to emphasize the relational component of smoking, the vulnerable hidden population of children in the smoking household, and how parental smoking can lead to family stress and negative health consequences to their children. Messages should include scenarios where youth feel distressed and trapped within their own homes. As well, messages that show concern for their health as well as the health of their siblings and parents may prove to be of value in getting the attention of parents and other adults who smoke. In a study by Nilsson and Emmelin, Swedish youth who smoked, felt strongly that parents had a duty to care and an obligation to do all they could to support their children not to start smoking: "*It's a parental duty*" [14]. Messages such as those voiced by youth in Nilsson and Emmelin’s study as well as those conveyed in the current study could potentially be powerful tools in smoking prevention and cessation programs.

This study’s findings are relevant to issues of childhood agency discussed by others [49]. Panel discussions with experts of tobacco control and community development revealed themes of children's levels of agency and their power in reducing their exposure to second-hand smoke in the home [49]. In fact, children were seen as potential agents of change and it was suggested that the voices of children towards their caregivers are potentially central in creating smoke-free homes. Youth are often afforded little opportunity to have their voices heard, and it is noteworthy that some youth in the study did not feel they could speak their mind to their parents about their parent's smoking habits. It is a matter of social justice in allowing and encouraging these youth voices to be heard.

### Strengths and limitations of the study

Using a qualitative research approach afforded the opportunity to understand youth from their frames of reference and experiences of reality. The findings reported here add to the existing literature by providing a richer description on youth’s experiences and beliefs about smoking. Limitations of our study included a sample that was primarily females (72%) and in the younger and middle age range of youth with only 17% of participants being 17 years and older. Fewer youth who are male and older could explain why we did not detect differences based on age or gender. Despite striving for a diverse sample, we were unable to obtain diversity in ethnic backgrounds and socioeconomic status. As well, most youth in this study did not smoke. Future work that accounts for limitations in the study’s sample might result in additional perspectives on the relational aspects of smoking that warrant tailoring smoking cessation programs and policies to address the differences. As well, longitudinal work is recommended, as the cross-sectional nature of our study did not afford us an understanding how perspectives of youth change over time.

## Conclusion

This study revealed that while youth often feel trapped by others smoking in their home and powerless to stop this behaviour, they took the position of educating, trying to influence, and ultimately protecting their parents regarding the harmful effects of smoking and second-hand smoke. The findings reinforce that more needs to be done in strengthening environments where youth can grow and flourish. Upholding the rights of youth to live in clean, healthy, and unpolluted environments is a right and fair public health policy. As one youth from our study assertively stated, parents and all adults should *“just stop smoking cause it could affect your kid’s life and yours!”*

## Competing interests

The authors declare that they have no competing interests.

## Authors’ contributions

Study design RLW; supervision of data collection RLW; analysis and interpretation of the data RLW, CK; manuscript preparation RLW, CK. All authors read and approved the final manuscript.

## Pre-publication history

The pre-publication history for this paper can be accessed here:

http://www.biomedcentral.com/1471-2458/12/965/prepub
